# De-escalation from ticagrelor to clopidogrel in acute coronary syndrome patients: a systematic review and meta-analysis

**DOI:** 10.1007/s11239-019-01860-7

**Published:** 2019-04-19

**Authors:** Dominick J. Angiolillo, Giuseppe Patti, Kam Tim Chan, Yaling Han, Wei-Chun Huang, Alexey Yakovlev, Dara Paek, Michael del Aguila, Shalini Girotra, Dirk Sibbing

**Affiliations:** 1 Department of Medicine, Division of Cardiology, University of Florida, ACC Building 5th floor, 655 West 8th Street, Jacksonville, FL 32209 USA; 2 Chair of Cardiology, University of L’Aquila, Via Camponeschi, 19, L’Aquila, Italy; 3 Director of Cardiac Catheterization and Interventional Laboratory, Consultant Cardiologist, Queen Elizabeth Hospital, 30 Gascoigne Rd, King’s Park, Hong Kong, Hong Kong; 4 Department of Cardiology, The General Hospital of Shenyang Military Region, 83 Wenhua Rd, Shenhe District, Shenyang City, Liaoning China; 5 Department of Critical Care Medicine, Kaohsiung Veterans General Hospital, No. 386 Dazhong 1st Road, Zuoying District, Kaohsiung City, Taiwan; 6 School of Medicine, National Yang-Ming University, Taipei, Taiwan; 7 Department of Physical Therapy, Fooyin University, Kaohsiung, Taiwan; 8 Almazov National Medical Research Centre, 12 Mayakovsky St, Saint Petersburg, Russia; 9 Doctor Evidence, 301 Arizona Ave #301, Santa Monica, CA USA; 10 Sanofi, General Medicine and Emerging Markets, 38 Beach Road, Singapore, Singapore; 11 Department of Cardiology, LMU München, Marchioninistraße 15, 81377 Munich, Germany; 12 DZHK (German Centre for Cardiovascular Research), Partner Site Munich Heart Alliance, Munich, Germany

**Keywords:** Acute coronary syndrome, De-escalation, Antiplatelet therapy, Meta-analysis

## Abstract

De-escalation from ticagrelor to clopidogrel in acute coronary syndrome (ACS) may occur for a variety of reasons, including side effects (bleeding and non-bleeding) and costs. This study sought to assess the prevalence of de-escalation from ticagrelor to clopidogrel and the occurrence of adverse clinical outcomes following de-escalation. We conducted a systematic review of clinical trials and real-world studies in ACS patients treated with ticagrelor. Real-world data on the prevalence of de-escalation during hospitalization or at discharge, after hospital discharge, and during the whole study period were included for meta-analysis. Major adverse cardiovascular events (MACE) and bleeding events occurring after de-escalation were also assessed. A total of 12 studies were eligible for meta-analysis of the prevalence of de-escalation. De-escalation from ticagrelor to clopidogrel therapy occurred with a mean prevalence of 19.8% [95% confidence interval (CI) 11.2–28.4%]. De-escalation occurred more frequently in-hospital or at discharge than after hospital discharge (23.7% vs. 15.8%). For assessment of clinical outcomes, a total of six studies were eligible for meta-analysis. Mean rate of MACE for patients with de-escalation was 2.1% (95% CI 1.1–4.1%) and the rate of major bleeding events was 1.3% (95% CI 0.4–4.5%). In conclusion, de-escalation commonly occurs in real-world practice. Although rates of major cardiovascular and bleeding events in this analysis were generally low, the profile of patients suitable for de-escalation, the impact of de-escalation on adverse clinical outcomes and how this is affected by the timing after index ACS warrants further large-scale investigation.

**Electronic supplementary material:**

The online version of this article (10.1007/s11239-019-01860-7) contains supplementary material, which is available to authorized users.

## Highlights

- With the availability of different oral P2Y_12_ receptor inhibitors, antiplatelet treatment strategies can be personalized based on individual patient risk for ischemic or bleeding complications.
- Recent clinical trial evidence demonstrate that an early and guided de-escalation strategy based on platelet function testing may be considered as an alternative treatment option for patients with acute coronary syndrome.
- Data from real world practice shows that non-guided de-escalation is common, although the clinical implications of this approach remain unknown.
- The profile of patients suitable for de-escalation, the impact of de-escalation on adverse clinical outcomes and how this is affected by the timing after index ACS warrants further large-scale investigation..

## Introduction

Current U.S. and European guidelines recommend dual antiplatelet therapy (DAPT) with aspirin plus a P2Y_12_ receptor inhibitor in patients with ACS [1–3]. The use of the newer generation P2Y_12_ inhibitors, prasugrel and ticagrelor, is generally recommended over clopidogrel in ACS patients because of their superior efficacy, albeit at the expense of increased bleeding [4–6]. The uptake of ticagrelor is superior to that of prasugrel among these due to its broader indications and less restrictions for use [6]. However, clopidogrel still remains a commonly prescribed agent worldwide due to its lower costs, tolerability and favorable benefit–risk ratio [7].

Switching between P2Y_12_ receptor inhibitors frequently occurs in real-world practice and de-escalation from a more potent to a less potent agent has become part of a stage-adapted therapy [8]. In this practice, providers use more potent P2Y_12_ inhibitors to increase protection from ischemic events in the early phase after ACS, and later switch to clopidogrel to reduce bleeding [9]. Indeed, while the ischemic benefit of the more potent P2Y_12_ inhibitors over clopidogrel persists over time, their greatest benefits are seen early, when the risk of ischemic complications is highest, while most hemorrhagic events with potent platelet inhibitors arise during chronic treatment [10, 11]. However, other reasons to de-escalate in clinical practice involve bleeding or non-bleeding side-effects (e.g., dyspnea) and costs [12, 13]. Although observational data suggest that a uniform de-escalation strategy early after an ACS may increase the risk of adverse events [14], recent randomized trial data from a smaller single-center trial suggests that when this occurs 4 weeks after hospital discharge, there is a reduced risk of bleeding complications without any trade-off in efficacy [15]. Considering conflicting data, larger sample sizes are needed to better define the clinical implications associated with de-escalation, including the assets and drawbacks of guided versus unguided de-escalation strategies [9, 15]. Despite the need for further investigations in the field, the recently released 2018 ESC/EACTS Guidelines on myocardial revascularization have included a new recommendation on guided DAPT de-escalation as a strategy that may be considered as an alternative treatment option for ACS patients [16].

We conducted a systematic review and meta-analysis with the following objectives: (1) to assess the prevalence and timing of de-escalation from ticagrelor to clopidogrel in patients with ACS, and (2) to assess the rate of clinical outcomes (ischemic events and bleeding) following de-escalation from ticagrelor to clopidogrel in patients with ACS.

## Materials and methods

### Data sources and searches

The literature search was performed in MEDLINE (via PubMed), Embase (via Ovid), and the Cochrane Central Register of Controlled Trials (via Wiley) from inception to April 18, 2017. References were limited to those published in the English language. Conference abstracts from the American College of Cardiology, European Society of Cardiology, American Heart Association, and the European Hematology Association from 2012 to 2017 were also included in the review. The complete search strategies are provided in the Supplemental Materials. The methods recommended by the Centre for Reviews and Dissemination, University of York were used [17].

### Study selection

A standardized review protocol was used to define the eligibility criteria for the search and screening of references using the PICO(TSS) framework, which outlines the population, interventions, comparators, outcomes, timing, setting, and study designs of interest (Table S1).

Eligibility criteria for studies on the prevalence of de-escalation included observational studies and registries on patient populations with ACS, including ST-elevation myocardial infarction (STEMI), non-ST-elevation myocardial infarction (NSTEMI), unstable angina (UA), who received treatment with ticagrelor. Outcomes included the prevalence rate of patients who switched from ticagrelor to clopidogrel, the time to switch or duration of initial ticagrelor therapy, and the reasons for de-escalation.

Eligibility criteria for studies on the clinical outcomes associated with de-escalation included clinical trials and observational studies in patients who received initial treatment with ticagrelor and subsequently switched to clopidogrel treatment. Efficacy outcomes included MI, stroke, stent thrombosis, and major adverse cardiovascular events (MACE), defined as the composite of cardiovascular death, MI, or stroke. Safety outcomes included any bleeding and major bleeding. Definitions for MACE and major bleeding reported in each study are provided in the Supplemental Materials.

### Data extraction and quality assessment

Study eligibility was determined by two reviewers (R.S. and K.S.) who independently screened the abstracts and full-text. Multiple publications from the same study were mapped as primary and companion publications. A third reviewer resolved discrepancies between two primary reviewers. Additional screening information is provided in the Supplemental Materials.

Data extraction was conducted using the Digital Outcome Conversion (DOC) Data version 2.0 software platform (Doctor Evidence, LLC, Santa Monica, CA, USA) and its universal electronic extraction form, based on a standardized data configuration protocol [18].

The Cochrane Collaboration tool was used to assess the risk of bias in randomized controlled trials (RCTs) [19], and the Newcastle–Ottawa Scale (NOS) was used to assess quality of non-randomized studies [20]. A description of the methods is available in the Supplementary Material.

### Statistical analysis

The prevalence and timing of de-escalation was analyzed by pooling the ticagrelor-treated cohorts to provide an overall estimate of the prevalence, or proportion, of patients switching to clopidogrel and the timing of de-escalation. When analyzing the clinical outcomes following de-escalation, a comparative analysis was preferred to make inferences regarding the choice between continuing initial ticagrelor therapy or de-escalation to clopidogrel; however, this was not feasible due to the lack of data reported for patients who remained on ticagrelor. Cohort analysis were performed instead and pooled groups that de-escalated from ticagrelor to clopidogrel therapy to determine the mean rate of outcomes, or proportion of patients experiencing the outcome, associated with de-escalation.

A random effects model using the restricted maximum likelihood (REML) method was used based on the observational study design and the heterogeneity observed between the studies [21]. The logit transformed proportions model were used for the analysis of clinical outcomes due to the probability of sparse data. The REML method was used to correct for the negative bias associated with the maximum likelihood (ML) method and is more robust to data outliers than ML estimators [22, 23]. Heterogeneity was assessed using the I^2^ statistic, with a value of I^2^ > 50% indicating significant heterogeneity. All analyses were performed using the R metaphor v2.0.0 package within the DOC Data version 2.0 software platform [24].

## Results

### Prevalence and timing of de-escalation

#### Summary of search results

The search for studies on the prevalence of de-escalation from ticagrelor to clopidogrel resulted in 1903 references. Following review, total of 12 observational studies met eligibility criteria and were included in meta-analysis [25–36]. The PRISMA flow diagram is presented in Figure S1A.

#### Study and group characteristics

A summary of the study characteristics is presented in Table 1, and summaries of group characteristics of the ticagrelor group across the included studies are presented in Tables S6A and S6B. Of the 12 observational studies included in the meta-analysis, seven were prospective and five were retrospective. Sample sizes for the ticagrelor group varied from 98 to 11,680 patients. Where reported, mean or median age spanned from 60 to 67.7 years of age. The proportion of females ranged from 22.5 to 36% across 11 studies reporting.

**Table 1:** Study characteristics of included studies for prevalence and timing of de-escalation

Study	Design	Country	Study N	Ticagrelor group (n)	Timing of de-escalation—with reasons^a^
Angeras et al. [25]	Retrospective Cohort	Sweden	1,04,012	Ticagrelor + aspirin (n = 11,680)	After discharge—NR
Biscaglia et al. [26]	Prospective Cohort	Italy	586	Ticagrelor^b^ (n = 586)	Varied—need for OAT, bleeding, intolerance, unwillingness, dyspnea
Coons et al. [27]	Retrospective Cohort	United States	8127	Ticagrelor^b^ (n = 309)	In-hospital—NR
Dehghani et al. [28]	Prospective Cohort	Canada	227	Ticagrelor^b^ (n = 227)	Varied—dyspnea, no coverage, significant bleeding
Dery et al. [29]	Prospective Cohort	Canada	2179	Ticagrelor^b^ (n = 242/241^c^)	At discharge—NR
Gaubert et al. [30]	Prospective Cohort	France	164	Ticagrelor^b^ (n = 164)	After discharge—NR
Green et al. [31]	Retrospective Cohort	Denmark	7016	Ticagrelor^b^ (n = 3159/3066^c^)	After discharge—NR
Hamid [32]	Retrospective Cohort	United Kingdom	98	Ticagrelor + aspirin (n = 98)	After discharge—NR
Harding et al. [33]	Prospective Cohort	New Zealand	992	Ticagrelor + aspirin (n = 243)	Varied—NR
Simeone et al. [34]	Retrospective Cohort	United States	15,788	Ticagrelor^b^ (n = 2323)	After discharge—NR
Wang et al. [35]	Prospective Cohort	China	417	Ticagrelor + aspirin (n = 99)	In-hospital or at discharge—NR
Zettler et al. [36]	Prospective Cohort	United States	8672	Ticagrelor^b^ (n = 226)	After discharge—NR

*NR* not reported, *OAT* oral anticoagulant

^a^Reasons reported in at least 10% of those who de-escalated are listed

^b^Study did not clearly specify whether all patients also received aspirin

^c^Number of patients enrolled/number of patients analyzed

#### Meta-analysis

**Fig. 1:**
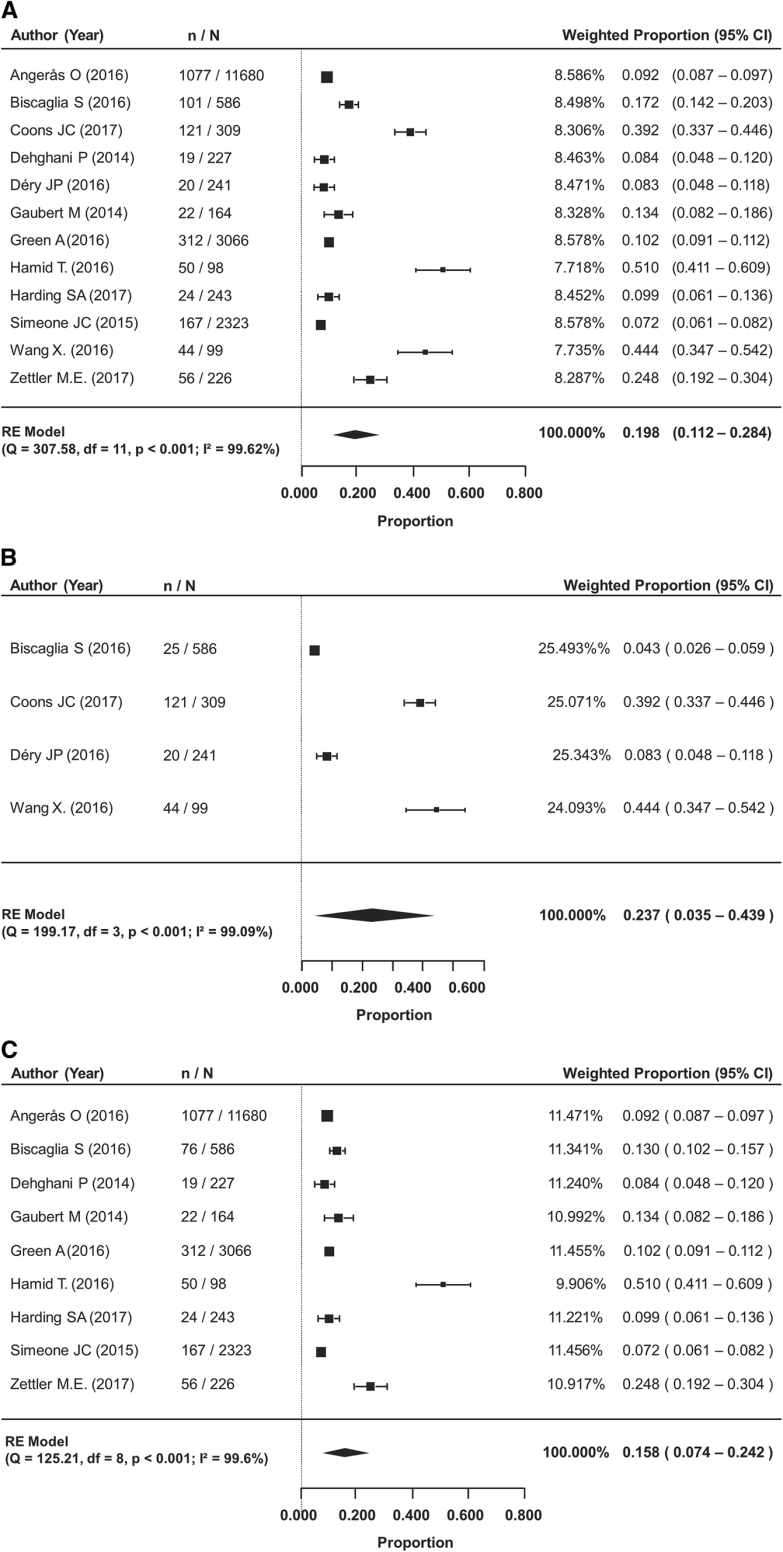
Prevalence of de-escalation from ticagrelor to clopidogrel. **a** De-escalation occurring during the entire study period (I^2^ = 99.62%); RE: Random Effects. **b** De-escalation occurring in-hospital or at discharge (I^2^ = 99.09%); RE: Random Effects. **c** De-escalation occurring after discharge (I^2^ = 99.60%); RE: Random Effects

The pooled prevalence of de-escalation from ticagrelor to clopidogrel among 12 studies (n = 19,262 analyzed) was 19.8% (95% confidence interval [CI] 11.2–28.4%). The meta-analysis was also sub-grouped by the timing of de-escalation: in-hospital or at the time of discharge, or after discharge. Rates reported from baseline through 1 year after the index event were included in the post-discharge subgroup analysis. De-escalation in-hospital or at discharge was reported in four studies, and after discharge in nine studies. The timing of de-escalation in each study and the reasons for switching reported by at least 10% of the patients are provided in Table 1. The prevalence of de-escalation in-hospital or at discharge was 23.7% (95% CI 3.5–43.9%), and 15.8% (95% CI 7.4–24.2%) after hospital discharge up to 1 year follow-up (Fig. 1b and c).

To analyze the precise timing of de-escalation, three studies (14,589 patients analyzed) were meta-analyzed that followed patients over 1 year (Figure S2). The mean duration of ticagrelor therapy before de-escalation to clopidogrel or discontinuation was 115 days (95% CI 81.2–148.4).

### Clinical outcomes associated with de-escalation

#### Summary of search results

The search for studies on the clinical outcomes associated with de-escalation from ticagrelor to clopidogrel treatment resulted in 1709 references. Following review, six studies met eligibility criteria and were included in meta-analysis [26, 32, 35, 37–39]. The PRISMA flow diagram is presented in Figure S1B.

#### Study and group characteristics

A summary of the study characteristics is presented in Table 2, and summaries of group characteristics of the ticagrelor group across the included studies are presented in Table S7A and 7B. Of the six studies included for meta-analysis, three were RCTs and three were observational (two prospective and one retrospective). All studies included a group taking ticagrelor followed by treatment with clopidogrel. Sample sizes for the ticagrelor followed by clopidogrel group varied from 44 to 265 patients. Where reported, mean or median age spanned from 62.1 to 72 years of age. The proportion of females ranged from 31.8% to 56% across 4 studies reporting.

**Table 2:** Study characteristics of included studies for clinical outcomes associated with de-escalation

Study	Design	Country	Study N	Ticagrelor group (n)	Timing of de-escalation—with reasons^a^	Follow-up duration
Biscaglia et al. [26]	Prospective Cohort	Italy	586	Ticagrelor followed by clopidogrel (n = 101)	Varied—need for OAT, bleeding, intolerance, unwillingness, dyspnea	12 months
Hamid [32]	Retrospective Cohort	United Kingdom	98	Ticagrelor + aspirin followed by clopidogrel + aspirin (n = 50)	3 months—NR	12 months
Wang et al. [35]	Prospective Cohort	China	417	Ticagrelor followed by clopidogrel (subgroup) (n = 44)	In-hospital or at discharge—NR	6 months
Motovska et al. [37]	RCT	Czech Republic	1230	Ticagrelor followed by clopidogrel (pooled with or without bolus) (n = 265)	Varied—economic	12 months
Pourdjabbar et al. [38]	RCT	Canada	60	Ticagrelor followed by clopidogrel (n = 60/57^b^)	Randomization—triple therapy, bleeding risk, cost, needing CABG, compliance concerns	30 days
Xu et al. [39]	RCT	China	114	Ticagrelor + aspirin followed by clopidogrel + aspirin (n = 57)	1 week—study protocol	Periprocedural

*CABG* coronary artery bypass graft, *NR* not reported, *OAT* oral anticoagulant, *RCT* randomized controlled trial

^a^Reasons reported in at least 10% of those who de-escalated are listed

^b^Number of patients enrolled/number of patients analyzed

#### Meta-analysis

**Fig. 2:**
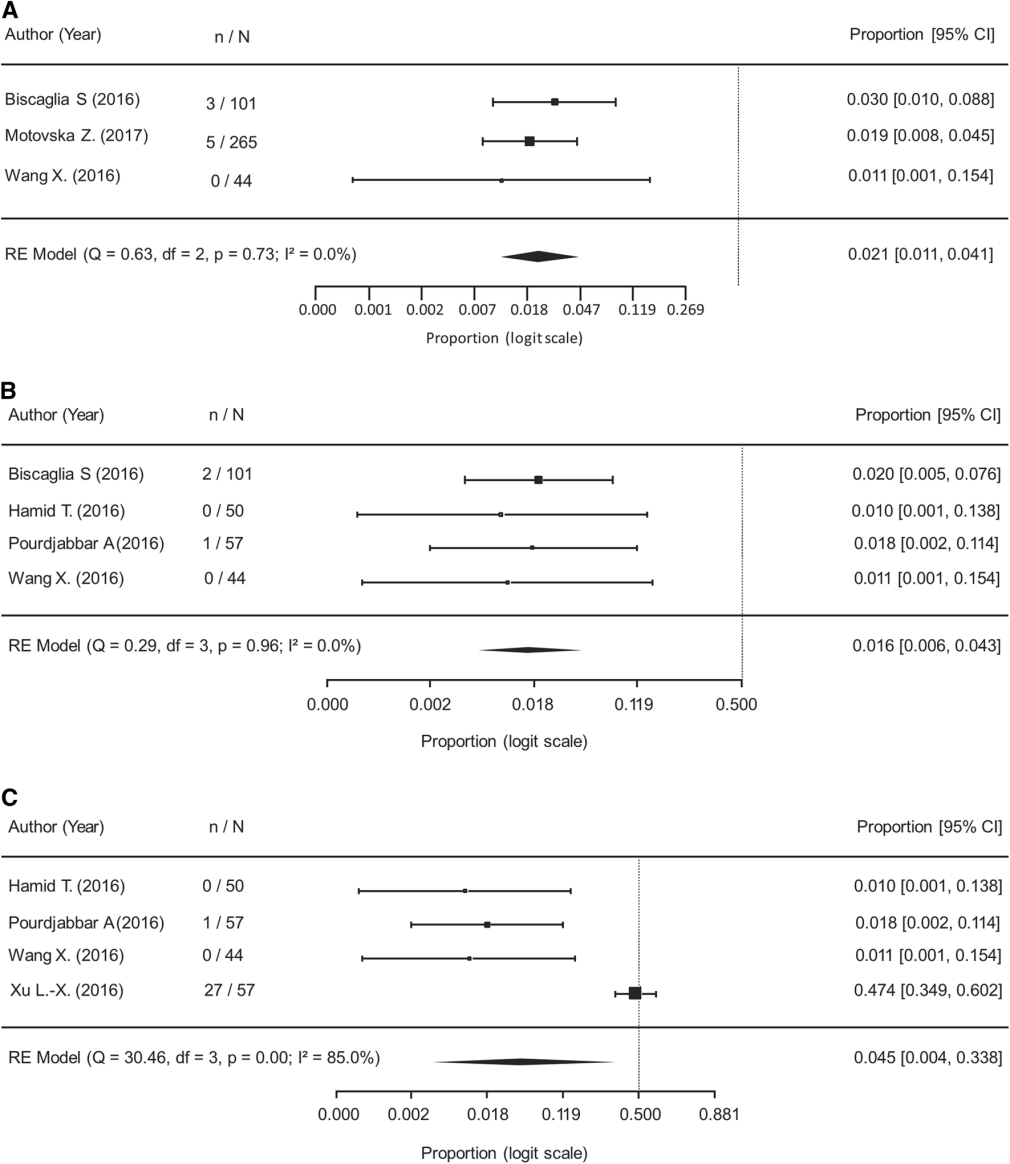
Cardiovascular outcomes following de-escalation from ticagrelor to clopidogrel. **a** Major Adverse Cardiovascular Events (I^2^ = 0.00%); RE: Random Effects. **b** Cardiovascular Mortality (I^2^ = 0.00%); RE: Random Effects. **c** Myocardial Infarction (I^2^ = 85.0%); RE: Random Effects

**Fig. 3:**
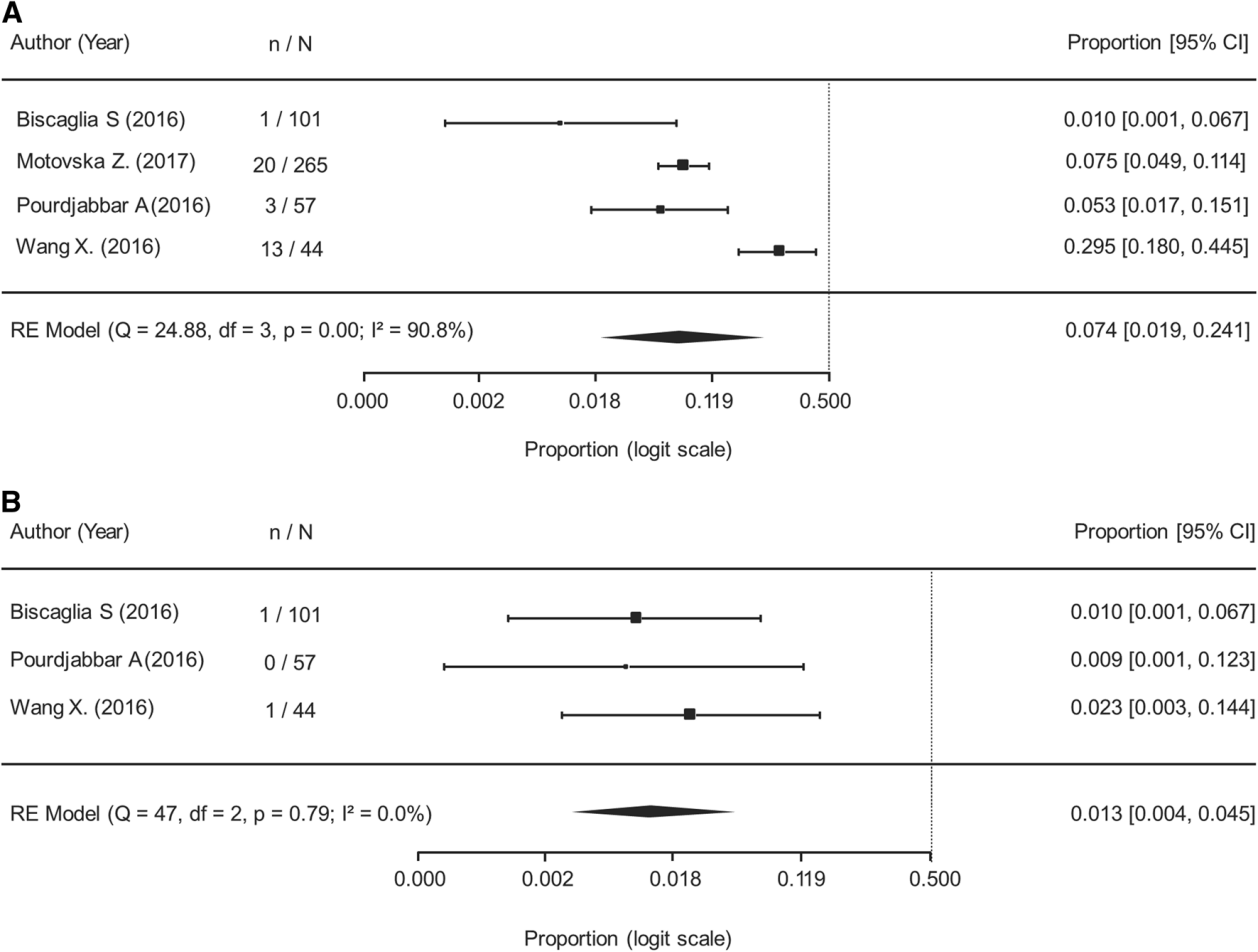
Bleeding events following de-escalation from ticagrelor to clopidogrel. **a** Any Bleeding (I^2^ = 90.8%); RE: Random Effects. **b** Major Bleeding (I^2^ = 0.00%); RE: Random Effects

When analyzing the safety and efficacy of de-escalation (574 patients analyzed), results of the meta-analysis showed the rate of MACE was 2.1% (95% CI 1.1–4.1%) during a mean follow-up duration of 10 months and with no observed heterogeneity (Fig. 2a). The rate of cardiovascular mortality was 1.6% (95% CI 0.6–4.3%) with no observed heterogeneity (Fig. 2b). The rate of MI was 4.5% (95% CI 0.4–33.8%) with significant heterogeneity observed (Fig. 2c). There were zero cases of stroke reported in 252 patients [26, 32, 35, 38] and one case of stent thrombosis reported in 202 patients who had available data following de-escalation from ticagrelor to clopidogrel [26, 35, 38]. The rate of any bleeding event was 7.4% (95% CI 1.9–24.1%) during a mean follow-up of 7.8 months and 1.3% (95% CI 0.4–4.5%) for major bleeding during a mean follow-up of 6.3 months (Fig. 3a and b, respectively).

## Discussion

To the best of our knowledge, this is the first systematic and dedicated meta-analysis assessing the prevalence, timing, and clinical outcomes of de-escalation from ticagrelor to clopidogrel therapy. In the absence of large observational studies or randomized clinical trials assessing this modality of de-escalation, the current study aimed to pool the relevant studies to offer insights into treatment patterns in the real-world and the clinical implications associated with such practice.

Our analysis showed that it is not infrequent for ACS patients to de-escalate to clopidogrel therapy following initial treatment with ticagrelor (pooled prevalence rate of 19.8%). We observed a higher prevalence rate of de-escalation occurring in-hospital or at discharge than after hospital discharge (23.7% vs. 15.8%). The rates of de-escalation in-hospital or at discharge were more variable across the studies, compared to studies reporting de-escalation after discharge; however, both results showed significant heterogeneity among the studies. Due to the fast offset action of ticagrelor, de-escalation to clopidogrel by standard loading dose regimens is recommended, regardless of the timing (acute vs chronic) of de-escalation except for patients with bleeding complications in whom de-escalation with a maintenance dose regimen may be considered [2, 12, 40]. The time to switch or duration of DAPT with ticagrelor, individual patient characteristics, and the specific reasons for de-escalation are underreported in the literature or not often documented in registries.

When assessing clinical outcomes after de-escalation, our analysis showed generally low rates across both ischemic outcomes and bleeding events, with no heterogeneity observed among studies for MACE and major bleeding. The observed aggregate event rates found in our review were comparable to those seen in clinical trials of de-escalation. Our results showed a rate of 2% for MACE (defined as CV mortality, MI, or stroke), 2% for CV mortality, and 1% for major bleeding. The TROPICAL-ACS study reported similar rates (3% and 1% and 1%, respectively) in patients with guided de-escalation from prasugrel to clopidogrel [9]. In the TOPIC study, CV death occurred in 0.3% and major bleeding in 0.3% of patients who were randomly assigned to downgrade from prasugrel/ticagrelor to clopidogrel [15]. Contrastingly, a different result is seen in observational data. In the SCOPE registry, a multicenter prospective non-randomized study that evaluated the incidence and short-term outcomes of switching oral P2Y_12_ inhibitors in ACS patients undergoing PCI, de-escalation was associated with an incidence of 20.4% for MACE and 3.8% for bleeding events [14]. In addition to the high-risk profile that patients from registries have compared with those from randomized clinical trials, these findings may be attributed to the fact that many patients switched therapy early after the index event, a time-frame in which patients are more vulnerable to thrombotic events and during which they could have benefited from more potent antiplatelet therapies.

There are several limitations regarding the findings of this study. The analysis was not performed using individual patient level data, thereby preventing adjustment of outcome data following de-escalation based on individual risk profile. Furthermore, studies did not report baseline risk variables for patients who de-escalated therapy. Duration of follow-up for outcomes, as well as the definitions for MACE and major bleeding outcomes, varied across studies. The prevalence analysis was conducted on data from observational studies, which have inherent source of bias, but do provide a more accurate assessment of prescribing behavior in the real-world setting. However, detailed information such as the timepoint of switch and patient outcomes following hospital discharge are not well reported in observational or registry data, thereby preventing landmark analyses for this review. Analyses of clinical efficacy and safety outcomes used a combination of data sourced from observational studies and RCTs. This poses challenges for determining the causal impact of de-escalation, but the inclusion of observational data may increase the generalizability of the results to real patient populations. As well, due to the limited data reported for patients remaining on ticagrelor therapy, a comparison with patients who de-escalated therapy could not be performed.

Based on the limitations and the considerable heterogeneity observed in some of our analyses, this study should be considered to be of exploratory nature. Dedicated and prospective studies are needed to provide evidence-based and practical recommendations on the optimal strategy to de-escalate DAPT therapy. These will inform on patient indicators that may benefit (or derive harm) from de-escalation, and whether the timing of de-escalation has an impact on clinical outcomes. Furthermore, further and large-scale randomized trials would allow an evaluation of de-escalation versus continuation of initial therapy. To this extent, a number of studies evaluating the use of genetic testing to guide antiplatelet treatment decisions making are currently ongoing and may add to the evidence of de-escalation guided by platelet function testing [9, 41, 42], and since the time of this review, there is also more observational data addressing the subject of pre-mature discontinuation of antiplatelet therapy [43]. Finally, defining the cost-effectiveness of de-escalation is warranted to better define its role in real-world practice. The role of platelet function and genetic testing guiding decision making on the choice of antiplatelet therapy to be used in patients undergoing PCI, has been recently revised in an updated international expert consensus document [44].

## Conclusions

Following index ACS treatment with ticagrelor, it is not uncommon for patients to de-escalate to clopidogrel. The analysis showed that rates of cardiovascular outcomes were generally low following de-escalation. However, further large-scale investigations are needed to appropriately examine the clinical implications of de-escalation on the risk of recurrent ischemic events and bleeding risk, as well as the appropriate timing to de-escalate patients in whom switching occurs.

## Electronic supplementary material

Below is the link to the electronic supplementary material.

Supplementary material 1 (DOCX 383 kb)
