# Supplementary material for: De-escalation from ticagrelor to clopidogrel in acute coronary syndrome patients: a systematic review and meta-analysis
Source: J Thromb Thrombolysis. 2019 Apr 19;48(1):1–10. doi: 10.1007/s11239-019-01860-7 (PMC6556170; doi:10.1007/s11239-019-01860-7)
Supplement: Supplementary file 1 — Supplementary material 1 (DOCX 383 kb) [file 11239_2019_1860_MOESM1_ESM.docx]

**SUPPLEMENTARY MATERIAL**

**Journal of Thrombosis and Thrombolysis**

**De-escalation from Ticagrelor to Clopidogrel in Acute Coronary Syndrome Patients: A Systematic Review and Meta-analysis**

Dominick J. Angiolillo*, Giuseppe Patti, Kam Tim Chan, Yaling Han, Wei-Chun Huang, Alexey Yakovlev, Dara Paek, Michael del Aguila, Shalini Girotra, Dirk Sibbing

**University of Florida, Department of Medicine, Division of Cardiology, Jacksonville, FL, USA (*dominick.angiolillo@jax.ufl.edu)

**STUDY SELECTION**

The complete list of included and excluded studies can be available upon request. Study selection was conducted by two reviewers (R.S. and K.S.) who independently screened the abstracts and full-text. A third reviewer (M.S) resolved discrepancies between two primary reviewers.

**RISK OF BIAS AND QUALITY ASSESSMENT**

## **Methods**

Cochrane tool is used to evaluate seven domains of bias: random sequence generation (selection bias), allocation concealment (selection bias), blinding of participants and personnel (performance bias), blinding of outcome assessment (detection bias), incomplete outcome data (attrition bias), selective reporting (reporting bias), and other sources of bias (other bias). The NOS assigns up to a maximum of 9 points (or stars) for the least risk of bias in 3 categories: 1) Selection of study groups (maximum 4 points); 2) Comparability of study groups (maximum 2 points); and 3) Exposure or Outcome ascertainment (maximum 3 points) for case–control or cohort studies, respectively. For the second item in the Outcome category, 2. “Was follow-up long enough for outcomes to occur”, a follow-up of 1 year or more was considered adequate. This length of follow-up has been used in several studies assessing the impact of DAPT therapy in ACS patients [1-3]. For the third item in the Outcome category, 3. “Adequacy of follow up of cohorts”, a follow-up rate of ≥90% or a description provided of those lost was considered adequate to assign a point.

## **Prevalence and timing of de-escalation**

A summary of the assessment of the 12 cohort studies is presented in **Table S3.** Of the studies included in the meta-analysis, eight studies scored at least a 6 out of a possible 9 points. All but one study scored 3 or 4 points in the Selection category, which can be attributed to their extensive multicenter databases. Gaubert et al. 2014 [4] was the one exception for poor Selection, scoring a 2 in this category as details were limited on how exposure and outcome assessments were made. Most studies lost points in the Comparability category, as baseline characteristics between groups were often quite imbalanced, especially with regard to age. Finally, in the Outcome category, seven studies scored a 2 or higher out of a possible score of 3. The remaining five studies scored 1 point in this category. Reasons for this low score can be attributed to an inadequate [4-7] or poorly described follow-up period length [8], which was much lower than our pre-specified acceptable length of 1 year.

## **Clinical outcomes associated with de-escalation**

A summary of the assessment of the three cohort studies included in the meta-analysis is presented in [**Table S4**](#_Table_9._Newcastle-Ottawa). Of these studies, two studies received 6 points as a final score covering the 3 categories, and one study received 5 points. All studies lost only one point in the Selection of cohorts category, as all included data from a single-center only, two did not use a standardized definition of a disease such as an ICD code when selecting their patients [7, 9], and no study explicitly stated that their outcome of interest was not present at the start of the trial. Overall, for the Comparability category judgement of bias was medium-low risk. Finally, in the Outcome category one study scored 2 points out of a possible 3 [9]. The other two studies [6, 7] lost points for inadequate follow-up period, and in one study [6], no description was provided on number of subjects lost to follow-up.

A summary of the assessment of the three RCTs included in the meta-analysis is presented in **Table S5**. In the trials included in the meta-analysis, the risk of selection bias was split between low or unclear for random sequence generation and allocation concealment. In two of the studies [10, 11], authors described the study as “randomized,” but insufficient details were provided on how randomization and allocation concealment were carried out. The risk of performance bias (pertaining to blinding of participants and personnel) and risk of detection bias (pertaining to blinding of outcome assessors) were mainly high as most study authors described the study as "open-label” [10, 12]. Attrition bias (pertaining to incomplete outcome data) was mainly low, as most authors included an intent-to-treat analysis with reasons provided for discontinuations [10, 12]. All trials had low risk of reporting bias (pertaining to selective reporting of outcomes) and other sources of bias. Assessment of Xu et al. 2016 [11] was based on a conference abstract; consequently, sufficient details were not provided to allow a comprehensive assessment and all domains were assessed as unclear risk of bias.**SUPPLEMENTAL FIGURES**

**Figure S1: PRISMA flow diagram.**

1. Prevalence and timing of de-escalation.


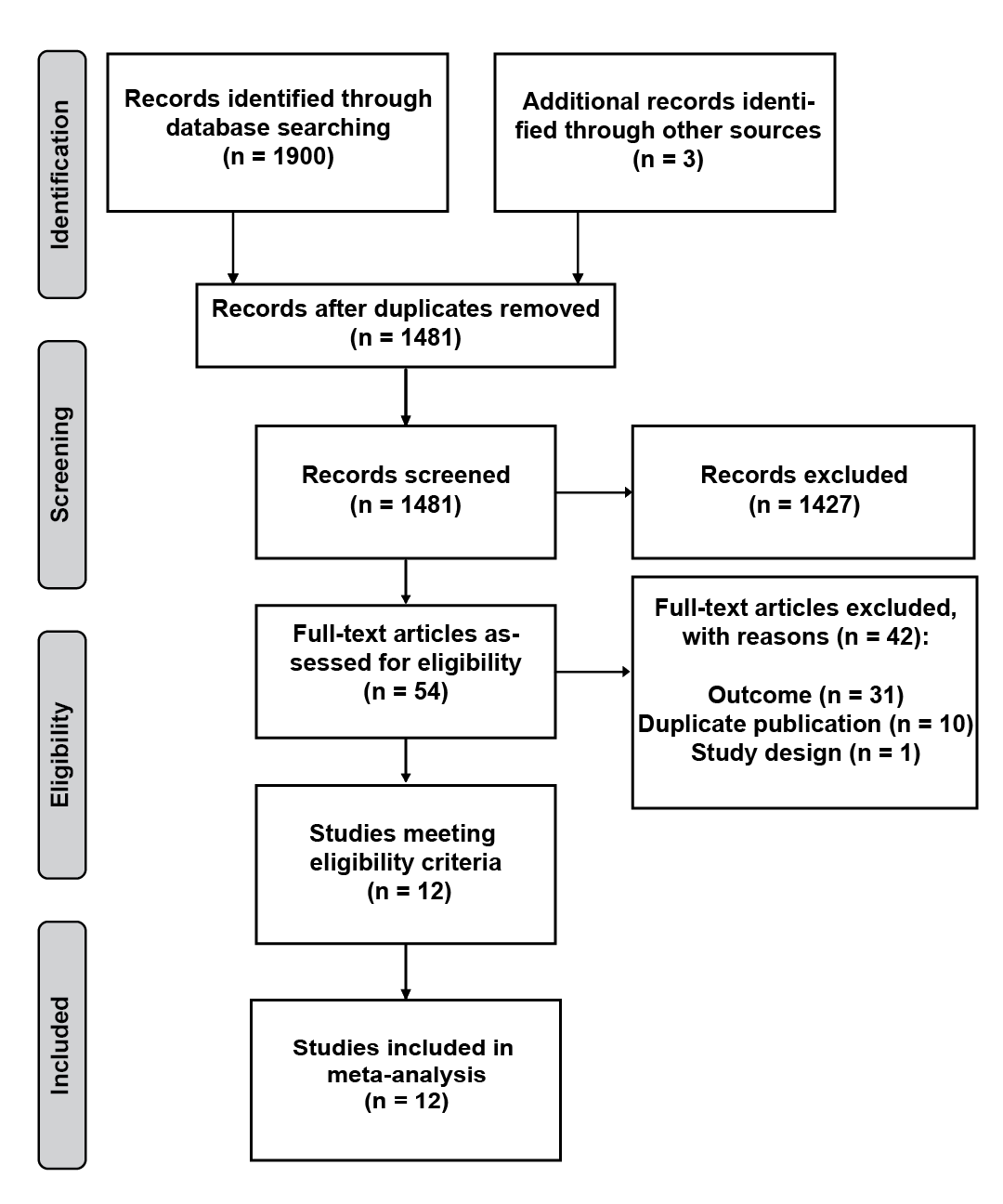


(B) Clinical outcomes following de-escalation.


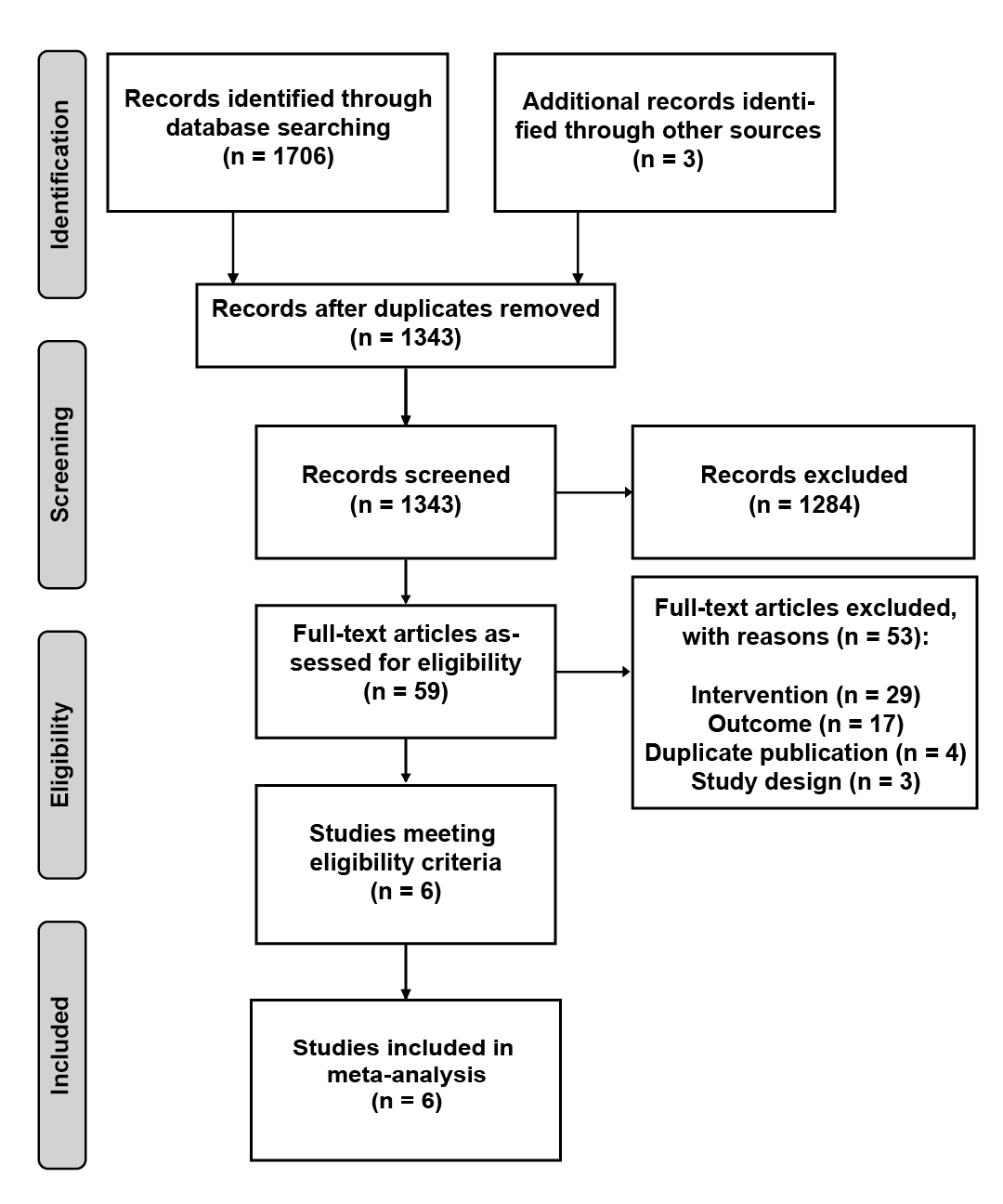


Note: Motovska et al. 2018 [10] was included after the original search conducted on April 18, 2017.

**Figure S2:** Mean duration (days) of ticagrelor therapy before de-escalation to clopidogrel or discontinuation.


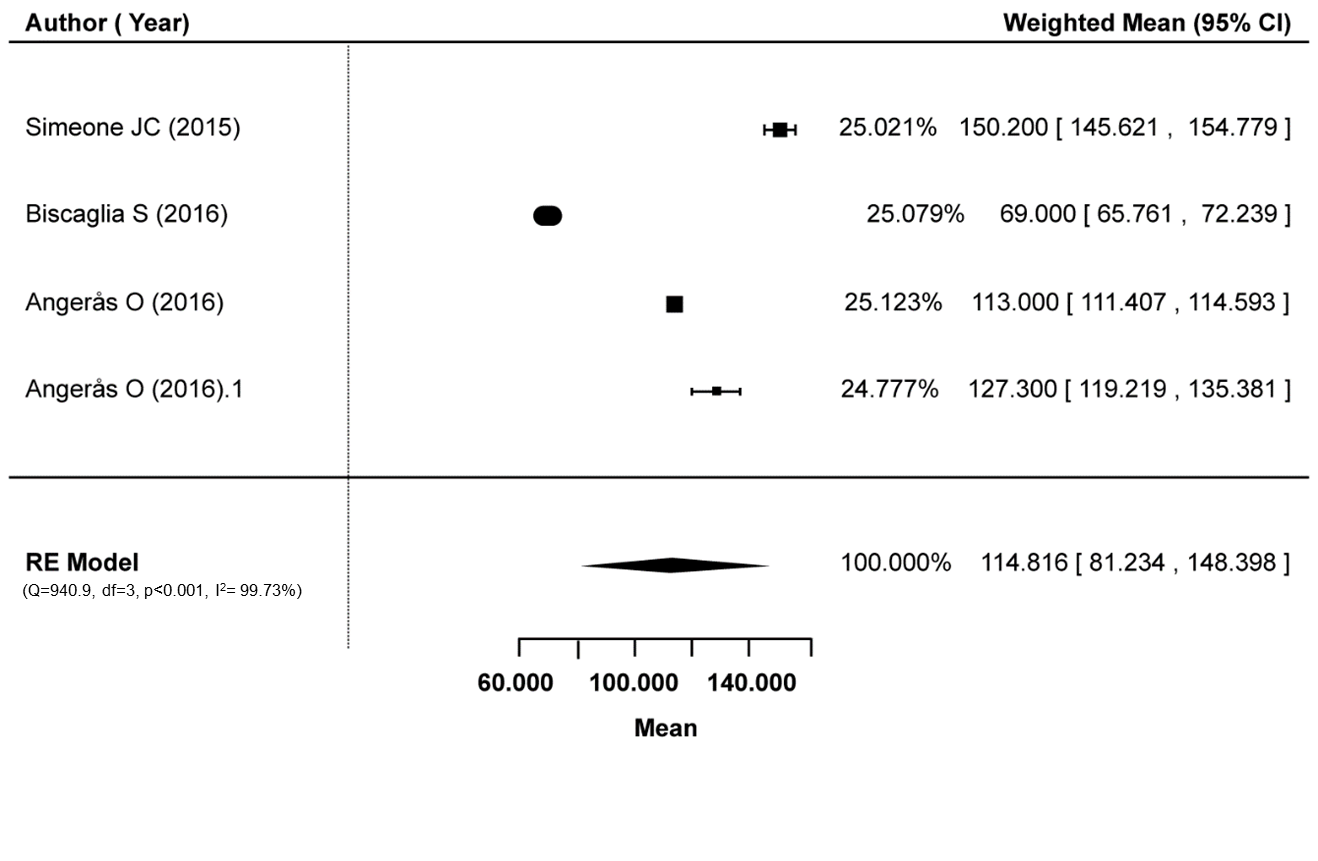


(I^2^ = 99.73%. Angerâs O (2016): angiographic cohort (N=11,045). Angerâs O (2016).1: non-angiographic cohort (N=635)); RE: Random Effects

# **SUPPLEMENTAL TABLES**

### Table S1: PICO(TSS) criteria for prevalence and clinical outcomes associated with timing of de-escalation.

| **Population** | - Acute coronary syndromes (UA / NSTEMI / STEMI) |
| --- | --- |
| **Interventions** | - Ticagrelor - Ticagrelor followed by switch to clopidogrel |
| **Comparators** | Any or no comparator |
| **Outcomes** | - Switch to clopidogrel - Timing of switch - Reasons for switching - Clinical outcomes - Major adverse cardiovascular events (MACE) - Mortality - Myocardial infarction - Stroke - Stent thrombosis - Bleeding |
| **Timing** | - No restriction to minimum treatment duration or follow-up - No restriction to outcome collected to certain time points |
| **Setting/context** | No restriction to study setting |
| **Study design** | - Observational studies - Clinical trials (for clinical outcomes only) |

NSTEMI: non-ST-elevation myocardial infarction, STEMI: ST-elevation myocardial infarction, UA: unstable angina.

### Table S2. Definitions for MACE and major bleeding reported by study

| **Study** | **MACE definition** | **Major bleeding definition** |
| --- | --- | --- |
| **Biscaglia et al. 2016** [9] | Cardiovascular death, MI, stroke or TIA | Bleeding academic research consortium (BARC) criteria, types 3-5 |
| **Hamid 2016** [6] | [Not included with MACE] All-cause mortality, cardiac mortality, MI, stroke, and target vessel revascularization | Definition not reported in study |
| **Wang et al. 2016** [7] | Definition 1: Cardiovascular death, non-fatal MI, non-fatal stroke  Definition 2: [Not included with MACE] Cardiovascular death, non-fatal MI, non-fatal stroke, defined or probable stent thrombosis, coronary revascularization, and re-hospitalization for unstable angina | Thrombosis in Myocardial Infarction (TIMI) criteria |
| **Motovska et al. 2018** [10] | Cardiovascular death, non-fatal MI, or stroke | Major bleeding not reported for subgroup of patients who de-escalated |
| **Pourdjabbar et al. 2016** [12] | [Not included with MACE] Cardiovascular death, recurrent MI, target vessel revascularization or stroke | Thrombosis in Myocardial Infarction (TIMI) criteria |
| **Xu et al. 2016** [11] | MACE not reported in study | Major bleeding not reported in study |

MACE: major adverse cardiovascular events, MI: myocardial infarction, TIA: transient ischemic attack

### Table S3. Newcastle-Ottawa quality assessment results for prevalence and timing of de-escalation.

| **Study** | **Included in MA? (Yes/No)** | **Study design** | **Selection** | **Comparability** | **Outcome** | **Final score** |
| --- | --- | --- | --- | --- | --- | --- |
| **Angeras et al. 2016** [13] | Yes | Cohort | ★★★★ | N/A | ★★★ | 7 |
| **Biscaglia et al. 2016** [9] | Yes | Cohort | ★★★ | ★ | ★★ | 6 |
| **Coons et al. 2017** [14] | Yes | Cohort | ★★★ | ★ | ★★★ | 7 |
| **Dehghani et al. 2014** [5] | Yes | Cohort | ★★★ | ★ | ★ | 5 |
| **Dery et al. 2016** [15] | Yes | Cohort | ★★★ | ★★ | ★★ | 7 |
| **Gaubert et al. 2014** [4] | Yes | Cohort | ★★ | N/A | ★ | 3 |
| **Green et al. 2016** [8] | Yes | Cohort | ★★★ | N/A | ★ | 4 |
| **Hamid 2016** [6] | Yes | Cohort | ★★★ | ★ | ★ | 5 |
| **Harding et al. 2017** [16] | Yes | Cohort | ★★★ | ★ | ★★★ | 7 |
| **Simeone et al. 2015** [17] | Yes | Cohort | ★★★★ | ★★ | ★★★ | 9 |
| **Wang et al. 2016** [7] | Yes | Cohort | ★★★ | ★★ | ★ | 6 |
| **Zettler et al. 2017** [18] | Yes | Cohort | ★★★★ | ★ | ★★★ | 8 |

N/A: not applicable, MA: meta-analysis. Maximum of 9 points (or stars) for the least risk of bias in 3 categories: 1) Selection of study groups (maximum 4 points); 2) Comparability of study groups (maximum 2 points); and 3) Exposure or Outcome ascertainment (maximum 3 points)

### Table S4. Newcastle-Ottawa quality assessment results for clinical outcomes associated with de-escalation.

| **Study** | **Included in MA? (Yes/No)** | **Selection** | **Comparability** | **Outcome** | **Final score** |
| --- | --- | --- | --- | --- | --- |
| **Biscaglia et al. 2016** [9] | Yes | ★★★ | ★ | ★★ | 6 |
| **Hamid 2016** [6] | Yes | ★★★ | ★ | ★ | 5 |
| **Wang et al. 2016** [7] | Yes | ★★★ | ★★ | ★ | 6 |

N/A: not applicable, MA: meta-analysis. Maximum of 9 points (or stars) for the least risk of bias in 3 categories: 1) Selection of study groups (maximum 4 points); 2) Comparability of study groups (maximum 2 points); and 3) Exposure or Outcome ascertainment (maximum 3 points)

### Table S5. Cochrane risk of bias assessment results for clinical outcomes associated with de-escalation.

| **Trial** | **Included in MA? (Yes/No)** | **Selection bias** | | **Performance bias** | **Detection bias** | **Attrition bias** | **Reporting bias** | **Other bias** |
| --- | --- | --- | --- | --- | --- | --- | --- | --- |
|  |  | **Random sequence generation** | **Allocation concealment** | **Blinding of participants and personnel** | **Blinding of outcome assessment** | **Incomplete outcome data** | **Selective reporting** | **Other sources of bias** |
| **Motovska et al. 2018** [10] | Yes | Unclear risk | Unclear risk | High risk | High risk | Low risk | Low risk | Low risk |
| **Pourdjabbar et al. 2016** [12] | Yes | Low risk | Low risk | High risk | High risk | Low risk | Low risk | Low risk |
| **Xu et al. 2016** [11] | Yes | Unclear risk# | Unclear risk# | Unclear risk# | Unclear risk# | Unclear risk# | Unclear risk# | Low risk# |

# Assessment was conducted on the conference abstract as the full-text was not available. MA: meta-analysis

### Table S6A. Group characteristics of included studies for prevalence and timing of de-escalation.

| **Study** | **Demographics** | | **Acute coronary syndrome characteristics** | | | **Underwent PCI, n (%)** |
| --- | --- | --- | --- | --- | --- | --- |
|  | **Mean/ Median age, years** | **Female, n (%)** | **UA, n (%)** | **NSTEMI, n (%)** | **STEMI, n (%)** |  |
| **Angeras et al. 2016** [13] | NR | NR | NR | NR | NR | NR |
| **Biscaglia et al. 2016** [9] | NR | 132 (22.53) | NR | NR | 217 (37.03) | 586 (100) |
| **Coons et al. 2017** [14] | NR | NR | NR | NR | NR | 309 (100) |
| **Dehghani et al. 2014** [5] | 62.2 | 78 (34.4) | NR | 80 (35.2) | 104 (45.81) | 209 (92.1) |
| **Dery et al. 2016** [15] | 60 | 58 (24) | NR | 76 (31.4) | 166 (68.6) | 242 (100) |
| **Gaubert et al. 2014** [4] | 61 | 41 (25) | 34 (20.5) | 79 (48.4) | 51 (31.1) | 164 (100) |
| **Green et al. 2016** [8] | NR | 1044 (33.05) | 0 (0) | NR | NR | 2238 (70.8) |
| **Hamid 2016** [6] | 67.7 | 35 (36) | NR | 98 (100) | NR | 73 (74.5) |
| **Harding et al. 2017** [16] | 61.5 | 59 (24.3) | NR | 209 (86) | 34 (14) | 156 (64.2) |
| **Simeone et al. 2015** [17] | 60.4 | 692 (29.8) | 482 (20.7) | 868 (37.4) | 944 (40.6) | 2323 (100) |
| **Wang et al. 2016** [7] | NR | 28 (28.28) | 62 (62.63) | 1 (1.01) | 20 (20.2) | 99 (100) |
| **Zettler et al. 2017** [18] | NR | 77 (34.07) | NR | NR | NR | 226 (100) |

NR: not reported, NSTEMI: non-ST-elevation myocardial infarction, STEMI: ST-elevation myocardial infarction, UA: unstable angina.

### Table S6B. Group characteristics of included studies for prevalence and timing of de-escalation, continued.

| **Study** | **Cardiovascular history** | | | | **Cardiovascular risk factors** | | | | **Comorbidity** |
| --- | --- | --- | --- | --- | --- | --- | --- | --- | --- |
|  | **PCI, n (%)** | **CABG, n (%)** | **MI, n (%)** | **Stroke/TIA, n (%)** | **Hypertension, n (%)** | **Diabetes, n (%)** | **Dyslipidemia/ Hyperlipidemia, n (%)** | **History of smoking, n (%)** | **CKD, n (%)** |
| **Angeras et al. 2016** [13] | NR | NR | NR | NR | NR | NR | NR | NR | NR |
| **Biscaglia et al. 2016** [9] | 95 (16.21) | 51 (8.7) | 171 (29.18) | NR | 400 (68.26) | 133 (22.70) | 297 (50.68) | 145 (24.74) | NR |
| **Coons et al. 2017** [14] | NR | NR | NR | NR | NR | NR | NR | NR | NR |
| **Dehghani et al. 2014** [5] | 42 (18.5) | 20 (8.8) | NR | NR | 117 (51.5) | 69 (30.4) | 107 (47.1) | NR | NR |
| **Dery et al. 2016** [15] | 97 (40.1) | 12 (5) | 109 (45.0) | 8 (3.3) | 127 (52.5) | 49 (20.2) | 135 (55.8) | 84 (34.71) | NR |
| **Gaubert et al. 2014** [4] | NR | NR | NR | NR | NR | NR | NR | NR | NR |
| **Green et al. 2016** [8] | NR | NR | NR | 102 (3.23) | NR | 419 (13.26) | NR | NR | NR |
| **Hamid 2016** [6] | NR | NR | NR | NR | 54 (55.10) | 20 (20.41) | 65 (66.33) | 40 (40.82) | NR |
| **Harding et al. 2017** [16] | NR | NR | 39 (16.4) | 10 (4.1) | 141 (58) | 33 (13.6) | 159 (65.4) | 55 (22.6) | 9 (3.7) |
| **Simeone et al. 2015** [17] | 84 (3.6) | 19 (0.8) | 137 (5.9) | 0 (0) | 1252 (53.9) | 668 (28.8) | 1166 (50.2) | NR | 152 (6.5) |
| **Wang et al. 2016** [7] | 25 (25.25) | 1 (1.01) | 11 (11.11) | 7 (7.07) | 59 (59.6) | 29 (29.29) | 35 (35.35) | 33 (33.33) | 2 (2.02) |
| **Zettler et al. 2017** [18] | NR | 17 (7.52) | 49 (21.68) | 23 (10.18) | NR | 68 (30.09) | NR | NR | NR |

NR: not reported, PCI: percutaneous coronary intervention, CABG: coronary artery bypass grafting, MI: myocardial infarction, TIA: transient ischemic attack, CKD: chronic kidney disease.

### Table S7A. Group characteristics of included studies for clinical outcomes associated with de-escalation.

| **Study** | **Demographics** | | **Acute coronary syndrome characteristics** | | | **Underwent PCI, n (%)** |
| --- | --- | --- | --- | --- | --- | --- |
|  | **Mean/ Median age, years** | **Female, n (%)** | **UA, n (%)** | **NSTEMI, n (%)** | **STEMI, n (%)** |  |
| **Biscaglia et al. 2016** [9] | 72 | 35 (35%) | NR | 58 (57% | 43 (43%) | 101 (100) |
| **Hamid 2016** [6] | NR | 28 (56%) | NR | 50 (100%) | NR | NR |
| **Wang et al. 2016** [7] | 62.1 | 14 (31.8%) | 33 (75%) | 1 (2.3%) | 4 (9.1%) | 44 (100) |
| **Motovska et al. 2018** [10] | NR | NR | NR | NR | NR | 265 (100) |
| **Pourdjabbar et al. 2016** [12] | 69.5 | 21 (35%) | NR | 21 (35%) | 39 (65%) | NR |
| **Xu et al. 2016** [11] | NR | NR | NR | NR | NR | 57 (100) |

NR: not reported, NSTEMI: non-ST-elevation myocardial infarction, STEMI: ST-elevation myocardial infarction, UA: unstable angina.

### Table S7B. Group characteristics of included studies for clinical outcomes associated with de-escalation, continued.

| **Study** | **Cardiovascular history** | | | | **Cardiovascular risk factors** | | | | **Comorbidity** |
| --- | --- | --- | --- | --- | --- | --- | --- | --- | --- |
|  | **PCI, n (%)** | **CABG, n (%)** | **MI, n (%)** | **Stroke/TIA, n (%)** | **Hypertension, n (%)** | **Diabetes, n (%)** | **Dyslipidemia/ Hyperlipidemia, n (%)** | **History of smoking, n (%)** | **CKD, n (%)** |
| **Biscaglia et al. 2016** [9] | 20 (20%) | 9 (9%) | 31 (31%) | NR | 69 (68%) | 23 (23%) | 52 (51%) | 29 (29%) | NR |
| **Hamid 2016** [6] | NR | NR | NR | NR | 22 (44%) | 11 (22%) | 32 (64%) | 21 (42%) | NR |
| **Wang et al. 2016** [7] | 14 (31.8%) | 1 (2.3%) | 6 (13.6) | 30 (68.2%) | 30 (68.2) | 16 (36.4) | 13 (29.5%) | 13 (29.5%) | 1 (2.3%) |
| **Motovska et al. 2018** [10] | NR | NR | NR | NR | NR | NR | NR | NR | NR |
| **Pourdjabbar et al. 2016** [12] | 15 (25%) | 4 (6.7%) | 21 (35%) | 8 (13.3%) | 40 (66.7%) | 24 (40%) | 25 (41.7%) | 28 (46.7%) | 4 (6.7%) |
| **Xu et al. 2016** [11] | NR | NR | NR | NR | NR | NR | NR | NR | NR |

NR: not reported, PCI: percutaneous coronary intervention, CABG: coronary artery bypass grafting, MI: myocardial infarction, TIA: transient ischemic attack, CKD: chronic kidney disease.

### Table S8: Search strategy for prevalence and timing of de-escalation via MEDLINE (via PubMed) (1946 to April 2017) conducted on April 18, 2017.

| **Set** | **Search strategy** | **Results** |
| --- | --- | --- |
| 1 | ("Acute Coronary Syndrome"[Mesh] OR acute coronary syndrome*[tiab] OR acs[tiab] OR "Myocardial Infarction"[Mesh] OR Myocardial Infarction[tiab] OR MI[ti] OR AMI[tiab] OR STEMI[tiab] OR NSTEMI[tiab] OR "Angina, Unstable"[Mesh] OR Unstable Angina[tiab] OR Myocardial preinfarction syndrome[tiab] OR Preinfarction angina[tiab] OR Angina at rest[tiab] OR Variant angina[tiab] OR Prinzmetals angina[tiab] OR ST Segment[tiab] OR ST Elevation[tiab] OR Non-st segment[tiab] OR non-segment[tiab] OR heart attack[tiab]) | 258509 |
| 2 | ("ticagrelor"[Supplementary Concept] OR ticagrelor[tiab] OR Brilinta[tiab] OR Brilique[tiab] OR AZD6140[tiab] OR AZD-6140[tiab]) | 1565 |
| 3 | (clopidogrel[Title/Abstract] OR "clopidogrel" [Supplementary Concept] OR plavix[tiab] OR iscover[tiab] OR SC25989C[tiab] OR SC-25989C[tiab] OR SC25990C[tiab] OR SC-25990C[tiab] OR SR-25989[tiab] OR SR25989[tiab] OR PCR4099[tiab] OR PCR-4099[tiab]) | 11808 |
| 4 | 2 AND 3 | 1080 |
| 5 | 1 AND 4 | 817 |
| 6 | ("Animals"[Mesh]) NOT (("Animals"[Mesh]) AND "Humans"[Mesh]) | 258509 |
| 7 | 5 NOT 6 | 813 |
| 8 | (((((((((("Review" [Publication Type]) OR "Comment" [Publication Type]) OR "Editorial" [Publication Type]) OR "Meta-Analysis" [Publication Type]) OR "In Vitro Techniques"[Mesh]) OR "News" [Publication Type]) OR "Newspaper Article" [Publication Type]) OR "Guideline" [Publication Type]) OR case report[Title])) OR ((((meta-analysis[Title]) OR meta-analyses[Title]) OR metaanalysis[Title]) OR metaanalyses[Title]) | 258509 |
| 9 | 7 NOT 8 | 405 |
| 10 | English[Language] | 22527074 |
| 11 | 9 AND 10 | 367 |

### Table S9: Supplemental P2Y12 search strategy for prevalence and timing of de-escalation via MEDLINE (via PubMed) (1946 to May 2017) conducted on May 19, 2017.

| **Set** | **Search strategy** | **Results** |
| --- | --- | --- |
| 1 | ("Acute Coronary Syndrome"[Mesh] OR acute coronary syndrome*[tiab] OR acs[tiab] OR "Myocardial Infarction"[Mesh] OR Myocardial Infarction[tiab] OR MI[ti] OR AMI[tiab] OR STEMI[tiab] OR NSTEMI[tiab] OR "Angina, Unstable"[Mesh] OR Unstable Angina[tiab] OR Myocardial preinfarction syndrome[tiab] OR Preinfarction angina[tiab] OR Angina at rest[tiab] OR Variant angina[tiab] OR Prinzmetals angina[tiab] OR ST Segment[tiab] OR ST Elevation[tiab] OR Non-st segment[tiab] OR non-segment[tiab] OR heart attack[tiab]) | 259517 |
| 2 | (Purinergic P2Y Receptor Antagonists [Pharmacological Action]) OR (Purinergic P2Y Receptor Agonists[Mesh] OR P2Y12 Purinoceptor Agonist*[tiab] OR Purinergic P2Y12 Receptor Agonist*[tiab] OR P2Y12 Purinoceptor Agonist*[tiab] OR Purinergic P2Y12 Receptor Agonist*[tiab] OR P2Y12 Purinoceptor Agonist*[tiab] OR Purinergic P2Y12 Receptor Agonist*[tiab] OR P2Y12 Purinoceptor Agonist*[tiab] OR P2Y12 Inhibitor*[tiab] OR P2Y12[tiab]) | 11318 |
| 3 | 1 AND 2 | 4329 |
| 4 | ((((((switch*[tiab] OR chang*[tiab] OR transition*[tiab] OR transfer*[tiab] OR move*[tiab] OR re-allocate*[tiab] OR reallocate*[tiab])) OR substitute*[tiab])) OR discontinu*[Title/Abstract])) OR "initially prescribed"[tiab] OR "initial prescription"[tiab] OR replac*[tiab] OR upgrad*[tiab] | 4122647 |
| 5 | 3 AND 4 | 655 |
| 6 | ("Animals"[Mesh]) NOT (("Animals"[Mesh]) AND "Humans"[Mesh]) | 4328003 |
| 7 | 5 NOT 6 | 653 |
| 8 | (((((((((("Review" [Publication Type]) OR "Comment" [Publication Type]) OR "Editorial" [Publication Type]) OR "Meta-Analysis" [Publication Type]) OR "In Vitro Techniques"[Mesh]) OR "News" [Publication Type]) OR "Newspaper Article" [Publication Type]) OR "Guideline" [Publication Type]) OR case report[Title])) OR ((((meta-analysis[Title]) OR meta-analyses[Title]) OR metaanalysis[Title]) OR metaanalyses[Title]) | 4149671 |
| 9 | 7 NOT 8 | 457 |
| 10 | (comparative study[pt] OR evaluation studies [pt] OR multicenter study[pt] OR validation studies[pt] OR observational study[pt] pragmatic clinical trial[pt] OR cohort studies[mh] OR cross-over studies[mh] OR follow-up studies[mh] OR retrospective studies[mh] OR prospective studies[mh] OR treatment outcome[mh] OR Controlled Before-After Studies[mh] OR observational[tiab] OR cohort*[tiab] OR follow-up[tiab] OR prospective[tiab] OR retrospective[tiab] OR cross-over[tiab] OR compar*[tiab] OR “this study”[tiab] OR “the current study”[tiab] OR “the present study”[tiab] OR “pilot study”[tiab] OR "naturalistic study"[tiab] OR "real world"[tiab] OR "single-arm"[tiab] OR "real world"[tiab] OR "real life"[tiab] OR case series[tiab]) OR case control studies[mh] OR case-control[tiab] OR cross sectional studies[mh] OR cross-sectional[tiab] OR model*[tiab] | 9352858 |
| 11 | 9 AND 10 | 392 |
| 12 | English[Language] | 22621859 |
| 13 | 11 AND 12 | 366 |

### Table S10: Search strategy for prevalence and timing of de-escalation via Embase (via Ovid) (1947 to April 2017) conducted on April 18, 2017.

| **Set** | **Search strategy** | **Results** |
| --- | --- | --- |
| 1 | *acute coronary syndrome/ or (acute coronary syndrome$ or acs).ti,ab. | 56499 |
| 2 | *myocardial infarction/ or (Myocardial Infarction or heart attack or AMI or STEMI or NSTEMI).ti,ab. or mi.ti. | 252829 |
| 3 | (Unstable Angina or Myocardial preinfarction syndrome or Preinfarction angina or Angina at rest or Variant angina or Prinzmetals angina).ti,ab. | 19248 |
| 4 | (ST Segment or ST Elevation or Non-st segment or non-segment).ti,ab. | 46205 |
| 5 | 1 or 2 or 3 or 4 | 307294 |
| 6 | *ticagrelor/ or (ticagrelor or Brilinta or Brilique or AZD6140 or AZD-6140).ti,ab. | 2981 |
| 7 | *clopidogrel/ or (clopidogrel or plavix or iscover or SC25989C or SC-25989C or SC25990C or SC-25990C or SR-25989 or SR25989 or PCR4099 or PCR-4099).ti,ab. | 21201 |
| 8 | 6 and 7 | 2102 |
| 9 | 5 and 8 | 1597 |
| 10 | (exp animal/ or nonhuman/) not exp human/ | 5900440 |
| 11 | 9 not 10 | 1589 |
| 12 | English.lg. | 25843046 |
| 13 | 11 and 12 | 1468 |
| 14 | (article or conference paper).pt. | 21930417 |
| 15 | 13 and 14 | 555 |
| 16 | (conference abstract or conference proceeding or "conference review").pt. | 2523965 |
| 17 | 13 and 16 | 559 |
| 18 | ("2012" or "2013" or "2014" or "2015" or "2016" or "2017").yr. | 7659166 |
| 19 | 17 and 18 | 496 |
| 20 | 15 or 19 | 1051 |
| 21 | remove duplicates from 20 | 958 |

### Table S11: Supplemental P2Y12 search strategy for prevalence and timing of de-escalation via Embase (via Ovid) (1947 to May 2017) conducted on May 19, 2017.

| **Set** | **Search strategy** | **Results** |
| --- | --- | --- |
| 1 | *acute coronary syndrome/ or (acute coronary syndrome$ or acs).ti,ab. | 56882 |
| 2 | *myocardial infarction/ or (Myocardial Infarction or heart attack or AMI or STEMI or NSTEMI).ti,ab. or mi.ti. | 253493 |
| 3 | (Unstable Angina or Myocardial preinfarction syndrome or Preinfarction angina or Angina at rest or Variant angina or Prinzmetals angina).ti,ab. | 19269 |
| 4 | (ST Segment or ST Elevation or Non-st segment or non-segment).ti,ab. | 46481 |
| 5 | 1 or 2 or 3 or 4 | 308235 |
| 6 | *purinergic P2Y receptor antagonist/ or (P2Y12 Purinoceptor Agonist$ or Purinergic P2Y12 Receptor Agonist$ or P2Y12 Purinoceptor Agonist$ or Purinergic P2Y12 Receptor Agonist$ or P2Y12 Purinoceptor Agonist$ or Purinergic P2Y12 Receptor Agonist$ or P2Y2 Purinoceptor Agonist$ or P2Y12 Inhibitor$ or P2Y12 Receptor Inhibitor* or P2Y12).ti,ab. | 48854 |
| 7 | (switch$ or chang$ or shift*$ or initially prescribed or initial prescription or upgrad$ or replac*).ti,ab. | 4121272 |
| 8 | 6 AND 7 | 998 |
| 9 | 5 and 8 | 425 |
| 10 | (exp animal/ or nonhuman/) not exp human/ | 5918835 |
| 11 | 9 not 10 | 418 |
| 12 | English.lg. | 25843046 |
| 13 | 11 and 12 | 372 |
| 14 | (article or conference paper).pt. | 22003148 |
| 15 | 13 and 14 | 220 |
| 16 | (conference abstract or conference proceeding or "conference review").pt. | 2553620 |
| 17 | 13 and 16 | 90 |
| 18 | ("2012" or "2013" or "2014" or "2015" or "2016" or "2017").yr. | 7768729 |
| 19 | 17 and 18 | 15 |
| 20 | 15 or 19 | 235 |
| 21 | remove duplicates from 20 | 209 |

### Table S12: Search strategy for clinical outcomes associated with de-escalation studies via MEDLINE (via PubMed) (1946 to April 2017) conducted on April 18, 2017.

| **Set** | **Search strategy** | **Results** |
| --- | --- | --- |
| 1 | ("Acute Coronary Syndrome"[Mesh] OR acute coronary syndrome*[tiab] OR acs[tiab] OR "Myocardial Infarction"[Mesh] OR Myocardial Infarction[tiab] OR MI[ti] OR AMI[tiab] OR STEMI[tiab] OR NSTEMI[tiab] OR "Angina, Unstable"[Mesh] OR Unstable Angina[tiab] OR Myocardial preinfarction syndrome[tiab] OR Preinfarction angina[tiab] OR Angina at rest[tiab] OR Variant angina[tiab] OR Prinzmetals angina[tiab] OR ST Segment[tiab] OR ST Elevation[tiab] OR Non-st segment[tiab] OR non-segment[tiab] OR heart attack[tiab])  AND  (“ticagrelor"[Supplementary Concept] OR ticagrelor[tiab] OR Brilinta[tiab] OR Brilique[tiab] OR AZD6140[tiab] OR AZD-6140[tiab])  AND  (clopidogrel[tiab] OR "clopidogrel" [Supplementary Concept] OR plavix[tiab] OR iscover[tiab] OR SC25989C[tiab] OR SC-25989C[tiab] OR SC25990C[tiab] OR SC-25990C[tiab] OR SR-25989[tiab] OR SR25989[tiab] OR PCR4099[tiab] OR PCR-4099[tiab])  NOT  (animals[mh] NOT humans[mh]) | 813 |
| 2 | AND eng[la] | 751 |

### Table S13: Search strategy for clinical outcomes associated with de-escalation studies via Embase (via Ovid) (1947 to April 2017) conducted on April 18, 2017.

| **Set** | **Search strategy** | **Results** |
| --- | --- | --- |
| 1 | *acute coronary syndrome/ or (acute coronary syndrome$ or acs).ti,ab. | 56499 |
| 2 | *myocardial infarction/ or (Myocardial Infarction or heart attack or AMI or STEMI or NSTEMI).ti,ab. or mi.ti. | 252829 |
| 3 | *unstable angina pectoris/ or (Unstable Angina or Myocardial preinfarction syndrome or Preinfarction angina or Angina at rest or Variant angina or Prinzmetals angina).ti,ab. | 20668 |
| 4 | (ST Segment or ST Elevation or Non-st segment or non-segment).ti,ab. | 46205 |
| 5 | 1 or 2 or 3 or 4 | 308007 |
| 6 | ticagrelor/ or (ticagrelor or Brilinta or Brilique or AZD6140 or AZD-6140).ti,ab. | 2981 |
| 7 | *clopidogrel/ or (clopidogrel or plavix or iscover or SC25989C or SC-25989C or SC25990C or SC-25990C or SR-25989 or SR25989 or PCR4099 or PCR-4099).ti,ab. | 21201 |
| 8 | 5 and 6 and 7 | 1597 |
| 9 | 8 not ((exp animal/ or nonhuman/) not exp human/) | 1589 |
| 10 | limit 9 to (english language and (article or conference paper)) | 555 |
| 11 | limit 9 to (english language and yr="2012 -Current" and (conference abstract or conference proceeding or "conference review")) | 496 |
| 12 | 10 or 11 | 1051 |
| 13 | remove duplicates from 12 | 952 |

### Table S14: Search strategy for clinical outcomes associated with de-escalation studies via Cochrane Central Register of Controlled Trials (CENTRAL) (via Wiley) (inception to April 2017) conducted on April 18, 2017.

| **Set** | **Search strategy** | **Results** |
| --- | --- | --- |
| 1 | [mh "Acute Coronary Syndrome"] or [mh "myocardial infarction"] or [mh "Angina, Unstable"] or (acute coronary syndrome* or acs or Myocardial Infarction or AMI or STEMI or NSTEMI or Unstable Angina or Myocardial preinfarction syndrome or Preinfarction angina or Angina at rest or Variant angina or Prinzmetals angina or ST Segment or ST Elevation or Non-st segment or non-segment or heart attack):ti,ab,kw | 27195 |
| 2 | (ticagrelor or Brilinta or Brilique or AZD6140 or AZD-6140):ti,ab,kw | 553 |
| 3 | (clopidogrel or plavix or iscover or SC25989C or SC-25989C or SC25990C or SC-25990C or SR-25989 or SR25989 or PCR4099 or PCR-4099):ti,ab,kw | 3445 |
| 4 | #1 and #2 and #3 | 313 |
| 5 | #4 not (pubmed or embase):an in Trials | 3 |

**REFERENCES**

1. Pfisterer M, Brunner-La Rocca HP, Buser PT, et al. Late clinical events after clopidogrel discontinuation may limit the benefit of drug-eluting stents: an observational study of drug-eluting versus bare-metal stents. J Am Coll Cardiol. 2006;48(12):2584-91.
2. van Werkum JW, Heestermans AA, Zomer AC, et al. Predictors of coronary stent thrombosis: the Dutch Stent Thrombosis Registry. J Am Coll Cardiol. 2009;53(16):1399-409.
3. Varenhorst C, Jensevik K, Jernberg T, et al. Duration of dual antiplatelet treatment with clopidogrel and aspirin in patients with acute coronary syndrome. Eur Heart J. 2014;35(15):969-78.
4. Gaubert M, Laine M, Richard T, et al. Effect of ticagrelor-related dyspnea on compliance with therapy in acute coronary syndrome patients. Int J Cardiol. 2014;173(1):120-1.
5. Dehghani P, Chopra V, Bell A, et al. Southern Saskatchewan Ticagrelor Registry experience. Patient Prefer Adherence. 2014;8:1427-35.
6. Hamid T, Zaman M, Rose S, Malik N. Switching of Ticagrelor to Clopidogrel at 3 Months in Patients
   Treated for Acute Coronary Syndrome; Single Centre Experience. Cardiovasc Pharm Open Access 5:194. 2016.
7. Wang X, Xi S, Liu J, et al. Switching between ticagrelor and clopidogrel in patients who underwent percutaneous coronary intervention: insight into contemporary practice in Chinese patients. Eur Heart J Suppl. 2016;18(Suppl F):F19-F26.
8. Green A, Pottegård A, Broe A, et al. Initiation and persistence with dual antiplatelet therapy after acute myocardial infarction: a Danish nationwide population-based cohort study. BMJ Open. 2016;6(5):e010880.
9. Biscaglia S, Campo G, Pavasini R, Tebaldi M, Tumscitz C, Ferrari R. Occurrence, causes, and outcome after switching from ticagrelor to clopidogrel in a real-life scenario: data from a prospective registry. Platelets. 2016;27(5):484-7.
10. Motovska Z, Hlinomaz O, Kala P, et al. 1-Year Outcomes of Patients Undergoing Primary Angioplasty for Myocardial Infarction Treated With Prasugrel Versus Ticagrelor. J Am Coll Cardiol. 2018;71(4):371-381.
11. Xu L-X, Chen K-Y, Liu T, Zheng X-T, Che J-J, Li G. GW27-e0058 Comparisons of loading doses of ticagrelor versus clopidogrel in preventing periprocedural myocardial infarction. J Am Coll Cardiol. 2016;68(16):C89-C90.
12. Pourdjabbar A, Hibbert B, Chong AY, et al. A randomised study for optimising crossover from ticagrelor to clopidogrel in patients with acute coronary syndrome. The CAPITAL OPTI-CROSS Study. Thromb Haemost. 2017;117(2):303-310.
13. Angerås O, Hasvold P, Thuresson M, Deleskog A, Öbraun O. Treatment pattern of contemporary dual antiplatelet therapies after acute coronary syndrome: a Swedish nationwide population-based cohort study. Scand Cardiovasc J. 2016;50(2):99-107.
14. Coons JC, Iasella CJ, Chanas T, et al. Comparative Effectiveness and Safety Analysis of Dual Antiplatelet Therapies Within an Integrated Delivery System. Ann Pharmacother. 2017;51(8):649-655.
15. Déry JP, Mehta SR, Fisher HN, et al. Baseline characteristics, adenosine diphosphate receptor inhibitor treatment patterns, and in-hospital outcomes of myocardial infarction patients undergoing percutaneous coronary intervention in the prospective Canadian Observational AntiPlatelet sTudy (COAPT). Am Heart J. 2016;181:26-34.
16. Harding SA, Holley A, Wilkins B, Fairley S, Simmonds M, Larsen PD. Contemporary antiplatelet therapy in acute coronary syndromes: are there differences in outcomes and discontinuation between clopidogrel and ticagrelor. Intern Med J. 2017;47(11):1298-1305.
17. Simeone JC, Molife C, Marrett E, et al. One-year post-discharge resource utilization and treatment patterns of patients with acute coronary syndrome managed with percutaneous coronary intervention and treated with ticagrelor or prasugrel. Am J Cardiovasc Drugs. 2015;15(5):337-50.
18. Zettler ME, Peterson ED, McCoy LA, et al. Switching of adenosine diphosphate receptor inhibitor after hospital discharge among myocardial infarction patients: Insights from the Treatment with Adenosine Diphosphate Receptor Inhibitors: Longitudinal Assessment of Treatment Patterns and Events after Acute Coronary Syndrome (TRANSLATE-ACS) observational study. Am Heart J. 2017;183:62-68.
